# Phenylalanine suppresses cell death caused by loss of fumarylacetoacetate hydrolase in *Arabidopsis*

**DOI:** 10.1038/s41598-022-17819-3

**Published:** 2022-08-08

**Authors:** Yihe Jiang, Qi Zhu, Hua Yang, Tiantian Zhi, Chunmei Ren

**Affiliations:** 1 College of Bioscience and Biotechnology, Hunan Agricultural University, Changsha, 410128 China; 2 College of Horticulture, Hunan Agricultural University, Changsha, 410128 China; 3 Crop Gene Engineering Key Laboratory of Hunan Province, Hunan Agricultural University, Changsha, 410128 China; 4 College of Life Sciences and Resources and Environment, Yichun University, Yichun, 336000 China

**Keywords:** Jasmonic acid, Plant molecular biology, Plant physiology

## Abstract

Fumarylacetoacetate hydrolase (FAH) catalyzes the final step of Tyrosine (Tyr) degradation pathway essential to animals and the deficiency of FAH causes an inborn lethal disease. In plants, a role of this pathway was unknown until we found that mutation of *Short-day Sensitive Cell Death1* (*SSCD1*), encoding *Arabidopsis* FAH, results in cell death under short day. Phenylalanine (Phe) could be converted to Tyr and then degraded in both animals and plants. Phe ingestion in animals worsens the disease caused by FAH defect. However, in this study we found that Phe represses cell death caused by FAH defect in plants. Phe treatment promoted chlorophyll biosynthesis and suppressed the up-regulation of reactive oxygen species marker genes in the *sscd1* mutant. Furthermore, the repression of *sscd1* cell death by Phe could be reduced by α-aminooxi-β-phenylpropionic acid but increased by methyl jasmonate, which inhibits or activates Phe ammonia-lyase catalyzing the first step of phenylpropanoid pathway, respectively. In addition, we found that jasmonate signaling up-regulates *Phe ammonia-lyase 1* and mediates the methyl jasmonate enhanced repression of *sscd1* cell death by Phe. These results uncovered the relation between chlorophyll biosynthesis, phenylpropanoid pathway and jasmonate signaling in regulating the cell death resulting from loss of FAH in plants.

## Introduction

Tyrosine (Tyr) degradation pathway includes the five-step enzymatic reactions, in which Tyr is first converted to 4-hydroxyphenylpyruvate by Tyr aminotransferase, then transformed into homogentisate by 4-hydroxyphenylpyruvate dioxygenase. Next, homogentisate dioxygenase catalyzes homogentisate to form maleylacetoacetate (MAA) that is isomerized by MAA isomerase to fumarylacetoacetate (FAA). Last, FAA is hydrolyzed by fumarylacetoacetate hydrolase (FAH) to fumarate and acetoacetate^1,2^. Tyr degradation is essential to animals, blockage of this pathway results in metabolic disorder diseases, in which the most severe disorder in humans is hereditary tyrosinemia type I (HT1), an inborn lethal disease caused by deficiency of FAH^3,4^. Loss of FAH in HT1 patients results in the accumulation of Tyr degradation intermediates including FAA and MAA, and then both would undergo spontaneous reduction to succinylacetoacetate followed by spontaneous nonenzymatic decarboxylation to succinylacetone (SUAC) toxic to cells and tissues^3^. Although the homologous genes putatively encoding homogentisate dioxygenase, MAA isomerase, and FAH were demonstrated to exist in plants^2,5^, the role of the Tyr degradation pathway in plants had been unclear until we cloned the *short-day sensitive cell death 1* (*SSCD1*) gene encoding an Arabidopsis putative FAH^6^. Loss of FAH in the *sscd1* mutant leads to spontaneous cell death under short-day conditions (SD)^6^ and the accumulation of SUAC^7^. SUAC inhibits the activity of δ-aminolevulinic acid dehydratase involved in Chlorophyll (Chl) biosynthesis, resulting in high production of the Chl biosynthesis intermediate protochlorophyllide (Pchlide) in the dark under SD^8^. The excessive accumulation of Pchlide induces the production of reactive oxygen species (ROS) upon light irradiation and thereby causes cell death^8^.

Phenylalanine (Phe) is catalyzed by Phe hydroxylase to Tyr and then degraded in animals, and the dietary restriction of Tyr and Phe can improve the condition of HT1 patients^4,9^. In plants, Phe could also be converted into Tyr and then via homogentisate, to plastoquinones and tocopherols, or to degradation of the aromatic ring^10^. However, Phe can carry through phenylpropanoid metabolism to produce secondary metabolites^10^. Phenylpropanoids are precursors to flavonoids, isoflavonoids, cumarins, and stilbenes, which have important functions in plant defense against pathogens and other predators, as UV light protectants, and as regulatory molecules in signal transduction and communication with other organisms^11^. In phenylpropanoid pathway, the first step is that Phe ammonia-lyase (PAL) catalyzes the deamination of Phe to give cinnamic acid^12,13^. The α-aminooxi-β-phenylpropionic acid (AOPP) is an inhibitor of PAL, treatment with AOPP could reduce the activity of PAL^14–16^. Jasmonates (JAs) including jasmonic acid, methyl jasmonate (MeJA), and other derivatives, are a basic class of plant hormones involved in plant growth, development, and responses to biotic and abiotic stresses^17–20^. Treatment with MeJA could increase the activity of PAL in plants such as Chinese bayberry^21^, wheat^22^, and tuberose^16^. The *PAL* gene expression is responsive to a variety of environmental stimuli including pathogen infection, wounding, nutrient depletion, UV irradiation, extreme temperatures, and other stress conditions^23–26^.

To investigate whether the uptake of Phe in plants increases the cell death caused by loss of FAH as it does in animals, in this study the *sscd1* mutant was treated with Phe and it was found that the death of *sscd1* seedlings is not increased but suppressed by Phe treatment. With further investigation, we found that Phe treatment promotes Chl biosynthesis and represses the up-regulation of ROS marker genes in the *sscd1* mutant. Furthermore, the repression of *sscd1* cell death by Phe is reduced by AOPP whereas enhanced by MeJA. In addition, MeJA up-regulates the *PAL1* gene and enhances the repression of *sscd1* cell death by Phe through JA signaling. Our study shows that Phe has different effects on the cell death caused by FAH loss in plants and animals and uncovers the relation between Chl biosynthesis, phenylpropanoid pathway, and JA signaling in regulating the cell death resulting from loss of FAH, which will help to further investigate the regulation of Tyr degradation, phenylpropanoid biosynthesis and the cell death in plants.

## Results

### Phe treatment suppresses the death of *sscd1* seedlings

**Figure 1:**
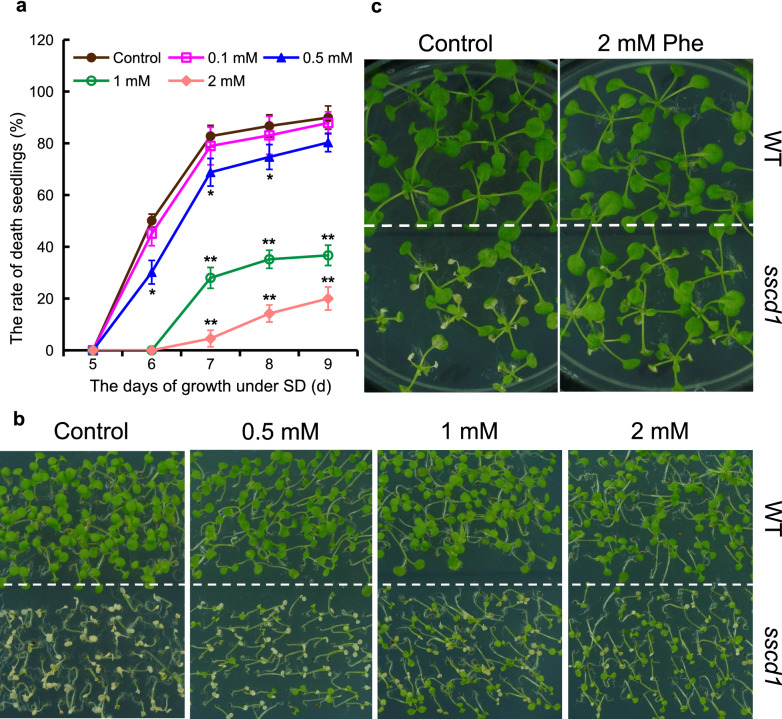
The death of *sscd1* seedlings was suppressed by treatment with Phe. (**a**) The death rate of *sscd1* seedlings which were grown in medium added without (control) or with 0.1, 0.5, 1, or 2 mM Phe under SD for 5–9 days. Error bars represent standard deviations (n > 30). The experiment was performed in three independent biological repeats. The single asterisk and double asterisks represent the significance of differences compared to the control (two-tailed Student’s t-test) at the level of *P* < 0.05 and *P* < 0.01, respectively. (**b**) The phenotype of WT and *sscd1* seedlings which were grown in medium added without (control) or with 0.5, 1 or 2 mM Phe under SD for 7 days. (**c**) The phenotype of WT and *sscd1* seedlings which were first grown in MS medium under LD for 7 days and transplanted to MS medium added without (control) or with 2 mM Phe for an additional 7-d growth under LD, and then transferred to SD for a 7-day growth. *Phe* phenylalanine; *SD* short day; *WT* wild type, Col-0; *LD* long day.

Since Phe could be converted into Tyr and then degraded in plants^10^, we wondered whether Phe treatment promotes the death of *sscd1* seedlings. To this end, the seeds of wild type and the *sscd1* mutant were plated on MS medium without or with 0.1, 0.5, 1 and 2 mM Phe and grown under SD. Unexpectedly, the death of *sscd1* seedlings treated with Phe was not increased, on the contrary, it was reduced (Fig. 1). When the medium was supplemented with 0.1 mM Phe, the rate of death seedlings was slightly reduced compared to that without Phe, however, it was significantly reduced as concentrations of Phe were increased (Fig. 1a). For example, on the 7th day, more than 80% of *sscd1* seedlings in the medium without Phe were dead, however, when the medium was supplemented with 0.5, 1, and, 2 mM Phe, the rates of death seedlings were only about 70%, 30%, and 5%, respectively (Fig. 1a). The phenotype of seedlings shown in Fig. 1b clearly displayed that Phe treatment reduced the death of *sscd1* seedlings. The *sscd1* seedlings are normal under long-day conditions (LD), however, once transferred to SD and grown for several days, some leaves of *sscd1* seedlings are dead and show bleaching, but the seedlings would not die^6^. To test whether the death of *sscd1* seedling leaves is also weakened by Phe, the seedlings growing under LD were treated with Phe and then transferred to SD. As shown in Fig. 1c, when *sscd1* seedlings growing in the medium with Phe under LD were transferred to SD, the extent of seedling leaf death was obviously attenuated. All these results indicated that Phe treatment suppresses the death of *sscd1* seedlings.

### The up-regulation of ROS marker genes in *sscd1* could be repressed by Phe treatment

**Figure 2:**
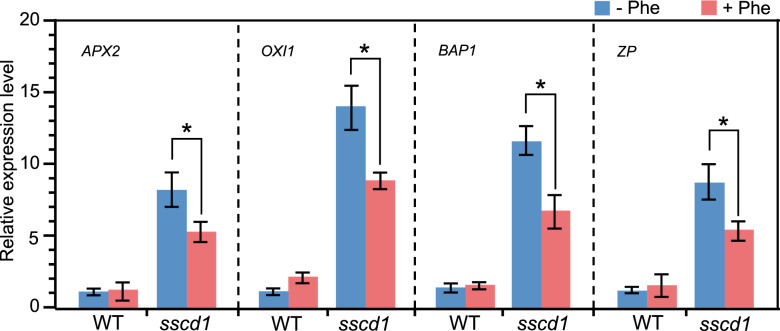
Expression of ROS marker genes in *sscd1* was down-regulated by treatment with Phe. Relative expression levels of *APX2, OXI1, BAP1* and *ZP* in WT and *sscd1* seedlings which were first grown in MS medium under LD for 7 days and transplanted to MS medium added without (−) or with (+) 2 mM Phe under LD for an additional 7-day growth, and then transferred to SD for a 3-d growth. *ACTIN2* expression was used as the internal control. Each value is the mean of three independent biological replicates ± standard deviation. An asterisk represents the significance of differences (two-tailed Student’s t-test) at the level of *P* < 0.05. *WT* wild type, Col-0; *Phe* phenylalanine; *LD* long day; *SD* short day.

Previously, we have speculated that the ROS resulting from excessive accumulation of Pchlide causes the *sscd1* cell death^8^. Because ROS marker genes such as *ascorbate peroxidase 2 (APX2)*^27^, *oxidative signal inducible 1 (OXI1)*^28,29^, *bonzai1-associated protein 1 (BAP1)*, and *transcription factor (ZP)*^30^ were up-regulated in the *sscd1* mutant^8,31^, we next investigated whether the repression of *sscd1* cell death by Phe is correlated with the expression level of these genes. As shown in Fig. 2, the expression levels of *APX2*, *OXI1*, *BAP1*, and *ZP* in the *sscd1* mutant were significantly increased compared to that in wild type, however, they were clearly reduced after Phe treatment. Therefore, the up-regulation of ROS marker genes in *sscd1* could be repressed by Phe treatment. Since the expression level of ROS marker genes is positively correlated to the content of ROS^27,29,30^, the repression of the up-regulation of ROS marker genes in *sscd1* by Phe treatment (Fig. 2) indicated the reduction of ROS after Phe treatment.

### Chl biosynthesis could be promoted by Phe treatment

**Figure 3:**
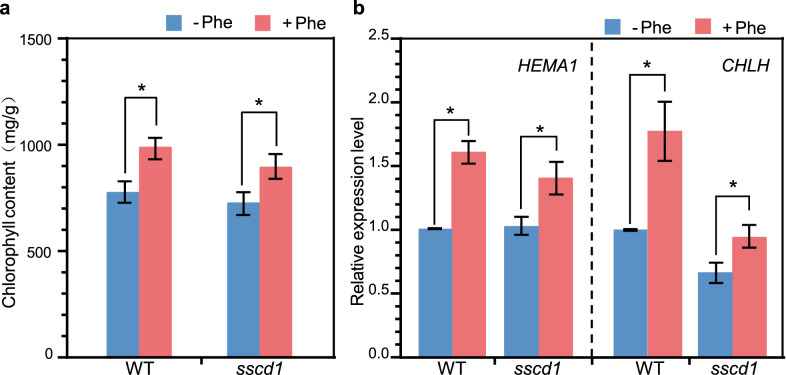
Chlorophyll synthesis was increased after Phe treatment. (**a**) The content of chlorophyll in WT and *sscd1* seedlings which were first grown in MS medium under LD for 7 days and transplanted to MS medium added without (−) or with (+) 2 mM Phe under LD for an additional 7-day growth, and then transferred to SD for a 3-day growth. (**b**) The relative expression levels of *CHLH* and *HEMA1* in WT and *sscd1* seedlings which were first grown in MS medium under LD for 7 days and transplanted to MS medium added without (−) or with (+) 2 mM Phe under LD for additional 7-d growth, and then transferred to SD for a 3-d growth. *ACTIN2* expression was used as the internal control. Each value is the mean of three independent biological replicates ± standard deviation. An asterisk represents the significance of differences (two-tailed Student’s t-test) at the levels of *P* < 0.05. *WT* wild type, Col-0; *LD* long day; *Phe* phenylalanine; *SD* short day.

Since the *sscd1* cell death is mediated by Chl biosynthetic pathway^8^, we next investigated whether the Phe treatment influences Chl biosynthesis. We first determined the content of Chl and found that it was increased after Phe treatment (Fig. 3a). In the Chl biosynthesis pathway, there are two pivotal control points, one is the formation of the initial precursor, δ-aminolevulinic acid, by glutamyl-tRNA reductase, and another is the metal-ion insertion step by Mg-chelatase^32–34^. In Arabidopsis, the *HEMA1* gene encodes the glutamyl-tRNA reductase and the *CHLH* gene encodes the H subunit of Mg-chelatase^32,34^. The transcriptional regulation of both *HEMA1* and *CHLH* could affect Chl biosynthesis^35^. Thus, we next tested whether the transcription of these genes changes after Phe treatment by RT-qPCR. As we expected, the expression levels of *HEMA1* and *CHLH* in both wild type and *sscd1* were also increased after Phe treatment (Fig. 3b). Therefore, Phe treatment promotes Chl biosynthesis.

### Repression of the *sscd1* cell death by Phe could be reduced by AOPP and enhanced by MeJA

**Figure 4:**
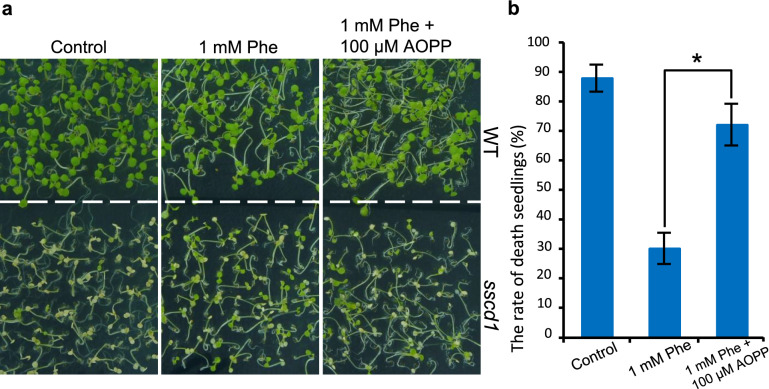
Treatment with AOPP reduced the inhibitory effect of Phe on the *sscd1* seedlings death. (**a**) The phenotype of 7-d-old wild-type WT and *sscd1* seedlings which were first grown in the medium added without (control) or with 1 mM Phe under SD for 2 days and then the seedlings in 1 mM Phe were treated with ddH_2_O (1 mM Phe) or 100 μM AOPP (1 mM Phe + 100 μM AOPP) for an additional 5-d growth. (**b**) The death rate of 7-d-old *sscd1* seedlings which were first grown in medium added without (control) or with 1 mM Phe under SD for 2 days and then the seedlings in 1 mM Phe were treated with ddH_2_O (1 mM Phe) or 100 μM AOPP (1 mM Phe + 100 μM AOPP) for an additional 5-d growth. Error bars represent standard deviations (n > 30). The experiment was performed in three independent biological repeats. An asterisk represents the significance of differences (two-tailed Student’s t-test) at the levels of p < 0.05. *WT* wild type, Col-0; *Phe* phenylalanine; *SD* short day.

Phe is a precursor of phenylpropanoid biosynthesis and PAL catalyzes the first step of this pathway^36^. If the repression of *sscd1* cell death by Phe is related to phenylpropanoid pathway, it would be changed by inhibition or activation of PAL. To confirm that, firstly, the *sscd1* seedlings were treated with AOPP, a potent inhibitor of PAL^15^, on the basis of Phe treatment. As we expected, treated with Phe and AOPP, the death *sscd1* seedlings were clearly increased compared to that treated with Phe alone (Fig. 4a). For example, the death rate of 7-d-old *sscd1* seedlings treated with 1 mM Phe was approximately 30%, however, when seedlings were treated with 1 mM Phe and 100 μM AOPP, it was increased to approximately 72% (Fig. 4b). These results indicated that the inhibition of PAL activity by AOPP could reduce repression of the *sscd1* cell death by Phe.

**Figure 5:**
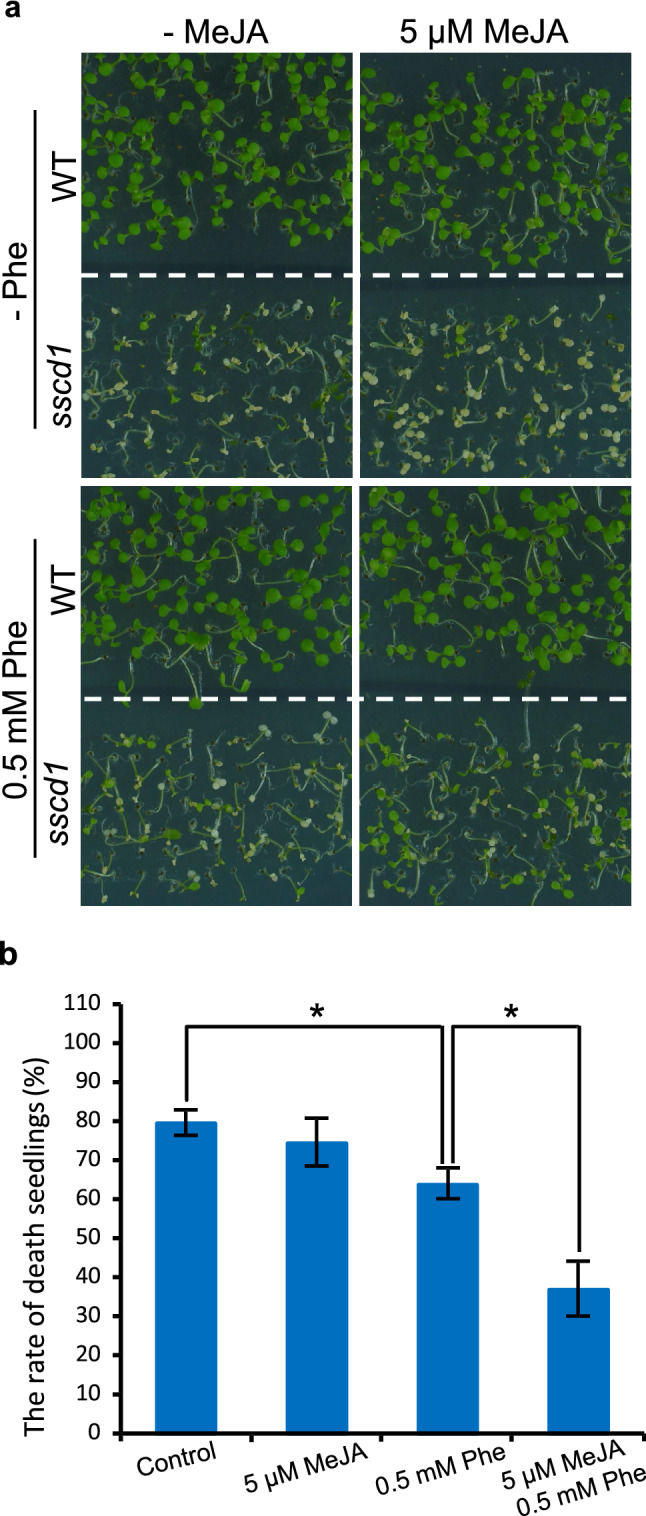
Treatment with MeJA enhanced the inhibitory effect of Phe on the *sscd1* seedlings death. (**a**) The phenotype of WT and *sscd1* seedlings which were grown in medium added without (−) or with 0.5 mM Phe or/and 5 μM MeJA under SD for 7 days. (**b**) The death rate of *sscd1* seedlings which were grown in medium added without (control) or with 5 μM MeJA or/and 0.5 mM Phe under SD for 7 days. Error bars represent standard deviations (n > 30). The experiment was performed in three independent biological repeats. An asterisk represents the significance of differences (two-tailed Student’s t-test) at the level of *P* < 0.05. *WT* wild type, Col-0; *Phe* phenylalanine; *MeJA* methyl jasmonate; *SD* short day.

Since the activity of PAL could be activated by MeJA^16^, we next investigated whether treatment with MeJA enhances the repression of *sscd1* cell death by Phe. As shown in Fig. 5a, in the absence of Phe, treatment with 5 μM MeJA did not distinctly affect the phenotype of both wild type and *sscd1* seedlings, however, after treatment with 5 μM MeJA and 0.5 mM Phe, the death seedlings of *sscd1* obviously reduced compared to those only treated with 0.5 mM Phe. The death rate of 7-d-old *sscd1* seedlings treated with 0.5 mM Phe was approximately 64% whereas it was less than 40% once treated with 5 μM MeJA and 0.5 mM Phe (Fig. 5b). Therefore, the activation of PAL activity by MeJA could enhance repression of the *sscd1* cell death by Phe.

### Treatment with MeJA causes the *COI1-*dependent up-regulation of *PAL1*

**Figure 6:**
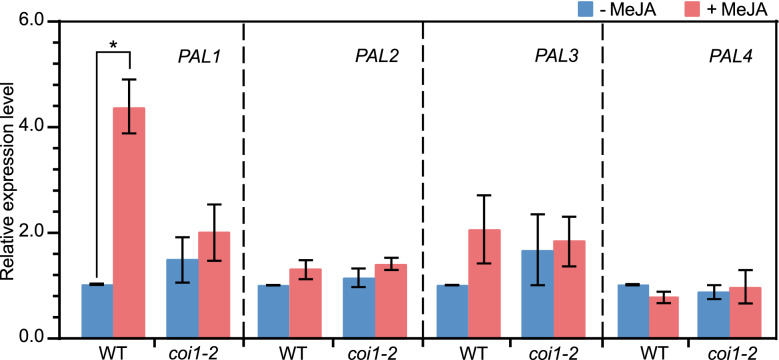
An analysis of expression of the *PAL* genes upon MeJA treatment by RT-qPCR. The relative expression levels of *PAL1, PAL2, PAL3* and *PAL4* in WT and *coi1-2* seedlings which were first grown in medium under LD for 12 days and removed to SD for a 3-d growth, and then treated with ddH_2_O (−) or 100 μM MeJA (+) for 1 day. *ACTIN2* expression was used as the internal control. Each value is the mean of three independent biological replicates ± standard deviation. An asterisk represents the significance of differences (two-tailed Student’s t-test) at the levels of *P* < 0.05. *WT* wild type, Col-0; *MeJA* methyl jasmonate; *LD* long day; *SD* short day.

In Arabidopsis, PAL is encoded by a small gene family including *PAL1*, *PAL2*, *PAL3*, and *PAL4*^26,37^. We next investigated whether treatment with MeJA affects some or all of these genes’ expression, and if so, is it dependent on COI1^17^, a JA receptor of JA signaling^38^? To this end, the seedlings of wild type and the *coi1-2* mutant, a *coi1* leaky mutant^39^, were treated with MeJA and the expression levels of *PAL1*, *PAL2*, *PAL3*, and *PAL4* were assessed by an analysis of RT-qPCR. As shown in Fig. 6, after treatment with MeJA, the expression level of *PAL1* was significantly increased in wild type but not in *coi1-2*. However, the expression levels of *PAL2*, *PAL3*, and *PAL4* were not significantly altered in both wild type and *coi1-2* after being treated with MeJA (Fig. 6). These results suggested that treatment with MeJA up-regulates *PAL1*, and this up-regulation is dependent on *COI1.*

### MeJA’s enhancement of Phe’s inhibition of *sscd1* cell death depends on *COI1*

**Figure 7:**
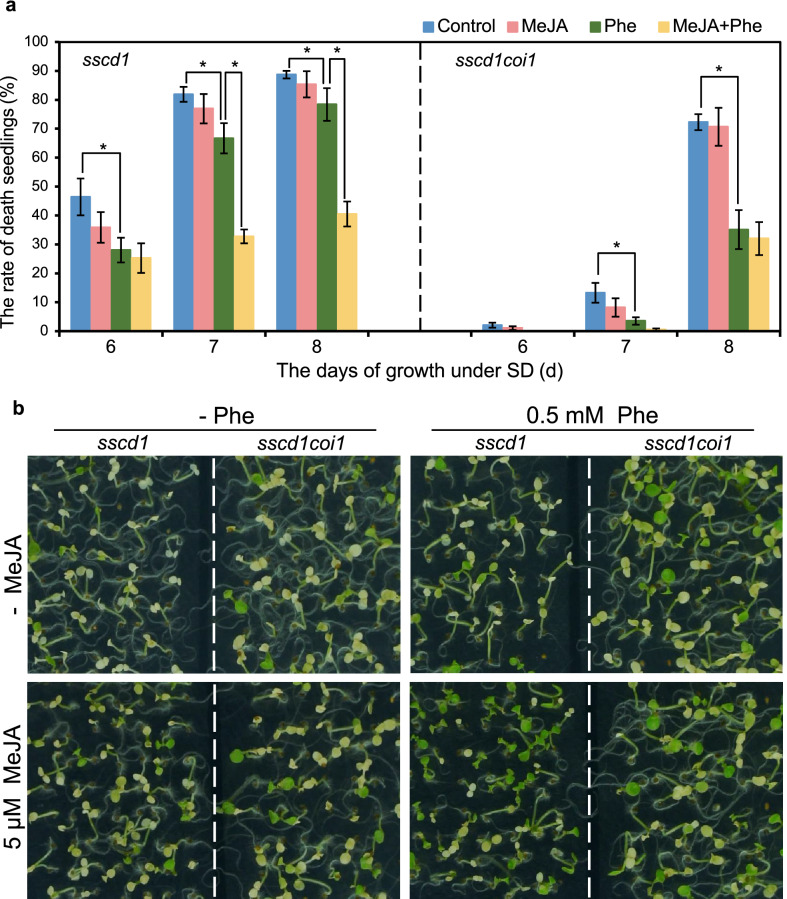
MeJA-increased Phe inhibition of *sscd1* seedlings death was dependent on *COI1*. (**a**) The death rate of *sscd1* (left) and *sscd1coi1* (right) seedlings which were grown in medium added without (Control) or with 5 μM MeJA or/and 0.5 mM Phe under SD for 6–8 days. Error bars represent standard deviations (n > 30). The experiment was performed in three independent biological repeats. An asterisk represents the significance of differences (two-tailed Student’s t-test) at the level of *P* < 0.05. (**b**) The phenotype of *sscd1* and *sscd1coi1* seedlings which were grown in medium added without (−) or with 0.5 mM Phe or/and 5 μM MeJA under SD for 8 days. *MeJA* methyl jasmonate; *Phe* Phenylalanine; *SD* short day.

Since treatment with MeJA enhanced the repression of *sscd1* cell death by Phe (Fig. 5) as well as up-regulated *PAL1* in dependence of *COI1* (Fig. 6), we next investigated whether the enhancement of MeJA on Phe in inhibiting the *sscd1* cell death is also dependent on *COI1*. The seeds of the *sscd1* single mutant and the *sscd1coi1* double mutant were plated on medium added without or with 0.5 mM Phe or/and 5 μM MeJA and grown under SD, and then the death seedlings were counted. As shown in Fig. 7a, the death rate of *sscd1coi1* seedlings was lower than that of *sscd1*, which is due to JA signaling positively regulating the *sscd1* cell death^31^. Phe treatment also significantly reduced the death rate of *sscd1coi1* seedlings (Fig. 7a, right), and that the reduction of seedling mortality was greater in *sscd1coi1* (Fig. 7a, right) than in *sscd1* (Fig. 7a, left), which is mainly resulted from that the blockage of JA signaling in *sscd1coi1* suppresses the *sscd1* cell death^31^ and Phe could also be degraded through the Tyr degradation pathway^10^. However, the death rate of *sscd1coi1* seedlings treated with MeJA and Phe was not significantly reduced compared to that treated with Phe alone (Fig. 7a, right), which is unlike in *sscd1* (Fig. 7a, left). From the Fig. 7b, we could see that MeJA treatment clearly enhances the repression of *sscd1* seedlings death by Phe, however, this enhancement was not obvious in *sscd1coi1* (Fig. 7b, right)*.* Therefore, the MeJA’s enhancement of Phe’s inhibition of *sscd1* cell death depends on *COI1*.

## Discussion

FAH catalyzes the final step of the Tyr degradation pathway and the deficiency of FAH in animals causes an inborn lethal disease, which was named HT1 in humans^1,3^. Phe could be converted to Tyr and then degraded in animals, and the dietary restriction of Tyr as well as Phe can improve the condition of HT1 patients^4,9^. In plants, the *SSCD1* gene encodes the Arabidopsis FAH and the mutation of *SSCD1* results in spontaneous cell death under SD^6^. Like as in animals, Phe could also be converted into Tyr in plants and then degraded^10^. However, in our study, the death of *sscd1* seedlings was not increased but repressed by Phe treatment (Fig. 1). So, why would Phe treatment repress the cell death resulting from loss of FAH in plants?

Previously, we demonstrated that the *sscd1* cell death is mediated by Chl biosynthesis^8^. The inhibition of the δ-aminolevulinic acid dehydratase activity by SUAC in the *sscd1* mutant influences Chl biosynthesis resulting in impairment of feedback inhibition of Chl biosynthesis from the light–dark transition under SD, which activates Chl biosynthesis and accumulation of Pchlide in the dark, and then upon re-illumination the excessive accumulation of Pchlide induces the mass production of ROS and thereby causes cell death^8^. The main form of ROS induced by Pchlide is singlet oxygen, which is unstable and difficult to be quantitatively detected^40^, however, it could be assessed through the analysis of the expression levels of some genes including *ZP* and *BAP1* activated specifically by singlet oxygen^30^. In this study, treatment of *sscd1* seedlings with Phe distinctly repressed the up-regulation of *ZP* and *BAP1* as well as other ROS marker genes such as *APX2* and *OXI1* (Fig. 2), In addition, we have tested hydrogen peroxide, another form of ROS, and found it was reduced in *sscd1* once treated with Phe (Fig. S1). Therefore, ROS is reduced by Phe treatment. Treatment with Phe could promote Chl biosynthesis (Fig. 3). The increase in Chl biosynthesis would restore to some extent the feedback inhibition of Chl biosynthesis in the *sscd1* mutant from the light–dark transition, as a result, reducing the accumulation of Pchlide in the dark and the production of ROS after subsequent exposure to light. Therefore, the increase of Chl biosynthesis should be one cause for the reduction of ROS and then the repression of cell death by Phe treatment.

In plants, Phe could be metabolized through the phenylpropanoid pathway to produce secondary metabolites, which plays an important role in plant against stress including UV-light, drought, and pathogen attack, due to their antioxidant function^10,11,26,41–43^. PAL catalyzes the first step of the phenylpropanoid pathway, which is a key step in phenylpropanoid biosynthesis^13,34,44^. The activity of PAL could be inhibited by AOPP and promoted by MeJA^16^. Treatment with AOPP prevents the increase in resistance to *B. cinerea* due to the application of external Phe^43^. In our study, the repression of *sscd1* seedlings death by Phe was reduced by AOPP (Fig. 4), however, it was enhanced by MeJA (Fig. 5), which suggested that the suppression of *sscd1* cell death by Phe is related to the phenylpropanoid pathway. Catechins, a class of flavonoids produced from Phe through phenylpropanoid pathway, have antioxidant activity^45^. In our study, treatment with catechins also suppressed the death of *sscd1* seedlings (Fig. S2). Since the secondary metabolites produced by Phe metabolism through the phenylpropanoid pathway have antioxidant function^42^, ROS could also be reduced by the metabolism of Phe through the phenylpropanoid pathway, which should be another important cause for the repression of *sscd1* cell death by Phe.

Previously, we found that JA signaling is involved in the *sscd1* cell death^31^. In the *sscd1* mutant, the accumulation of SUAC results in the generation of ROS, which induces cell death as well as JA synthesis^31^. JA up-regulates the Tyr degradation pathway, producing more SUAC, which promotes cell death^31^. Once JA signaling is broken by mutation of *COI1* encoding a JA receptor^38^, the up-regulation of Tyr degradation pathway by JA is eliminated, reducing the production of SUAC, as a result, the *sscd1* cell death is repressed^31^. MeJA is an activator of PAL catalyzing the first step of phenylpropanoid pathway^16^. In this study, MeJA treatment markedly increased the expression level of *PAL1* in wild type but not in the *coi1-2* mutant (Fig. 6), indicating that JA signaling can up-regulates the phenylpropanoid pathway through activating *PAL1*. The repression of *sscd1* cell death by Phe could be enhanced by MeJA treatment in the *sscd1* mutant (Figs. 5 and 7) but not in the *sscd1coi1* mutant (Fig. 7), which suggested that MeJA treatment enhances Phe inhibition of the *sscd1* cell death through JA signaling. Therefore, JA has a dual regulatory effect on the *sscd1* cell death. On the one hand, JA up-regulates the Tyr degradation pathway, promoting the *sscd1* cell death, on the other hand, JA up-regulates the phenylpropanoid pathway, inhibiting the *sscd1* cell death. For this reason, the death of *sscd1* seedlings was not increased by MeJA treatment, it seemed to decrease slightly (Figs. 5 and 7), which suggested that the effect of MeJA treatment on the *sscd1* cell death through the phenylpropanoid pathway might be greater than that through Tyr degradation pathway.

In addition, we observed that with Phe treatment alone the decrease of death seedlings in *sscd1coi1* is greater than that in *sscd1* at 8 days (Fig. 7). One main reason for that should be that the blockage of JA signaling in *sscd1coi1* decreases the seedlings death because JA signaling positively regulates the *sscd1* cell death^31^. Another main reason for that should be that Phe could also be degraded through the Tyr degradation pathway^10^, inducing production of more ROS and subsequent cell death, however, blockage of JA signaling in *sscd1coi1* could suppress the *sscd1* cell death^31^. As a result, with Phe treatment alone the suppression of the seedlings death in the double mutant *sscd1coi1* should be more obvious than that in the single mutant *sscd1*.

**Figure 8:**
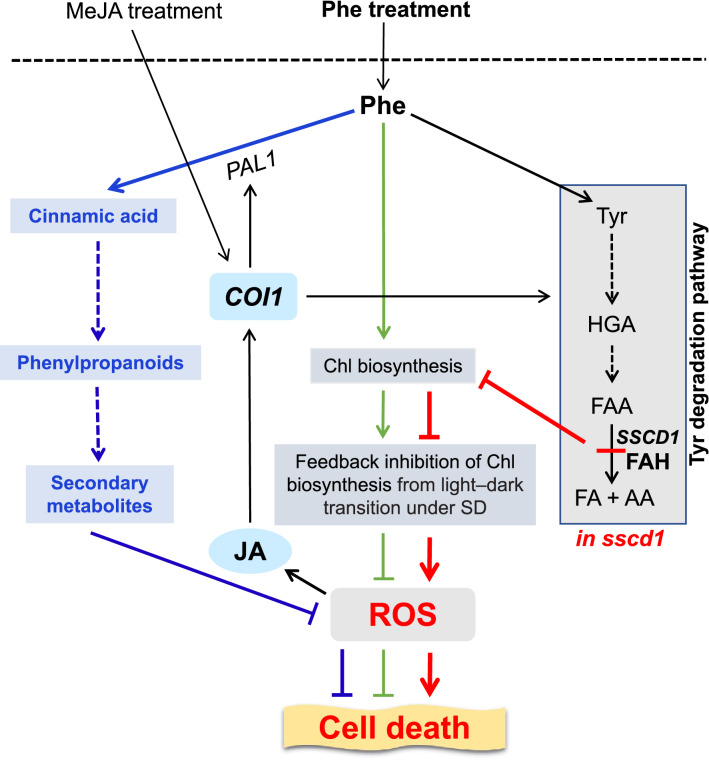
The possible mechanisms by which Phe treatment represses the *sscd1* cell death. The *SSCD1* gene encodes FAH catalyzing the last step of Tyr degradation pathway. Disruption of FAH in *sscd1* reduces Chl biosynthesis, which impairs the feedback inhibition of Chl biosynthesis from light–dark transition under SD, leading to the accumulation of ROS and then cell death (red lines and arrows). Phe treatment promotes Chl biosynthesis, which increases the feedback inhibition of Chl biosynthesis from light–dark transition under SD, reducing ROS and subsequent cell death (green arrows and lines). Meanwhile, Phe treatment activates phenylpropanoid pathway, which reduces ROS and subsequent cell death (blue arrows and lines). In the *sscd1* mutant, ROS induces cell death and the JA production. JA up-regulates Tyr degradation pathway through *COI1*, promoting cell death, however, it also up-regulates *PAL1* through *COI1*, which activates phenylpropanoid pathway, repressing cell death. The effect of MeJA treatment on *sscd1* cell death through phenylpropanoid pathway might be greater than that through Tyr degradation pathway, resulting in that MeJA treatment enhances the repression of *sscd1* cell death by Phe. Arrows indicate induction or positive regulation, whereas lines indicate repression or negative regulation. Arrows with dashed lines indicate multiple steps. *FAH* fumarylacetoacetate hydrolase; *Chl* chlorophyll; *SD* short day; *ROS* reactive oxygen species; *Phe* phenylalanine; *JA* jasmonates; *MeJA* methyl jasmonate; *HGA* homogentisate; *FAA* fumarylacetoacetate; *FA* fumarate; *AA* acetoacetate.

In conclusion, although Phe can be degraded through the Tyr degradation pathway, unlike in animals, Phe treatment does not increase the cell death resulting from loss of FAH in plants, instead, it represses the cell death. A possible mechanism for the repression of *sscd1* cell death by Phe treatment can be described as follows (Fig. 8). Loss of FAH in the *sscd1* mutant results in a decline of Chl biosynthesis, which impairs the feedback inhibition of Chl biosynthesis from light–dark transition under SD, leading to the accumulation of ROS and then cell death. Phe treatment, on the one hand, promotes Chl biosynthesis, increasing the feedback inhibition of Chl biosynthesis from light–dark transition under SD, and on the other hand, activates the phenylpropanoid pathway, both of which reduce ROS and subsequent cell death. In addition, in the *sscd1* mutant ROS induces cell death as well as JA synthesis. JA signaling up-regulates the Tyr degradation pathway, promoting the *sscd1* cell death, however, it also up-regulates *PAL1* which activates the phenylpropanoid pathway, repressing the *sscd1* cell death. Since the effect of MeJA treatment on the *sscd1* cell death through the phenylpropanoid pathway might be greater than that through the Tyr degradation pathway, the repression of *sscd1* cell death by Phe could be enhanced by MeJA treatment.

## Methods

### Plant materials and growth conditions

*Arabidopsis thaliana* L. ecotype Columbia-0 (Col-0) was obtained from the Arabidopsis Biological Resource Center (ABRC; Ohio State University, Columbus, OH, USA) and the mutants used in this study are in Col-0 background. The *sscd1* mutant^6^ was isolated by Han et al. in our laboratory. The *coi1-2* mutant^39^ was kindly provided by Professor Xie (Tsinghua University, Beijing, China) and the *sscd1coi1* double mutant^31^ was generated through a cross of *sscd1* with *coi1-2* by Zhou et al. in our laboratory. Experimental research on plants including the collection of plant material was performed in accordance with relevant institutional, national, and international guidelines and legislation.

Seeds were surface sterilized with 20% (v/v) chlorine bleach containing 0.1% (v/v) Triton X-100 for 10 min and washed three to five times with sterile water, then plated on Murashige & Skoog medium supplemented with 1% (m/v) sucrose and 0.7% (w/v) agar (pH 5.8) (MS). The different concentrations of Phe (SIGMA) were added to the MS medium. Plates were chilled at 4 °C in darkness for 3 days and then transferred to a growth chamber with LD (16-h light/8-h dark) or SD (8-h light/16-h dark) under 150 μmol photons m^−2^ s^−1^, controlled temperature (22 ± 2 °C).

For RT-qPCR analysis (Figs. 2, 3b) and determination of Chl content (Fig. 3a), 1-week-old seedlings growing under LD were transplanted onto MS added without or with Phe and grown under LD for an additional 1 week’s growth, and then transferred to SD for three days growth. Since the cell death of *sscd1* seedlings occurs on the 4th day after transferred from LD to SD^6^, the seedlings were harvested at the end of the third day's light for the determination of Chl content or harvested at 2 h light after three days for RT-qPCR analysis.

### Determination of the dead seedlings

A dead seedling is one for which all leaves were completely bleached. The rate of seedling death was calculated as the percentage of dead seedlings. The number of seedlings counted was approximately 100, and the experiment was performed in three independent biological repeats.

### RT-qPCR analysis

Total RNA was isolated using TRIZOL reagent (Life Technologies, https://www.thermofisher.com/us/en/home/brands/life-technologies.html). After incubation with DNase I (RNase Free, Thermo Fisher Scientific, https://www.thermofisher.com/) at 37 °C for 30 min and then at 65 °C for 10 min to remove genomic DNA, the RNA concentration and purity were measured spectrophotometrically using OD260/OD280 and OD260/OD230 ratios (ND-1000, NanoDrop, THERMO FISHER SCIENTIFIC). Complementary DNA was synthesized from the mixture of oligo-dT primers and random primers using a ReverTraAce qPCR RT kit (perfect real time) according to the manufacturer’s instructions (Toyobo, http://www.toyobo-global.com/).

RT-qPCR was performed in 96-well blocks using a SYBR qPCR mix (Roche, https://lifescience.roche.com/) with a Bio-Rad CFX Connect™ Real-Time PCR detection system (http://www.biorad.com/) following the manufacturer’s instructions. The RT-qPCR amplifications were performed under the following conditions: initial denaturation at 95 °C for 10 min, followed by 40 cycles of 95 °C for 15 s and 60 °C for 60 s. The primers of genes tested by RT-qPCR are listed in Table 1, and *ACTIN2* was used as an internal control. The gene expression for each sample was calculated on three analytical replicates, and the relative expression was quantified using the 2^−△△Ct^ method. The experiment was performed in three independent biological repeats. The significance of differences between datasets was evaluated using the two-tailed Student’s t-test.

**Table 1:** Primers of genes tested by real-time quantitative PCRs.

Gene	Forward primer	Reverse primer
*APX2* (AT3G09640)	5*'*-ACAAAGTTGAGCCACCTCCT-3*'*	5*'*-AAGGTGTGTCCACCAGACAA-3*'*
*OXI1* (AT3G25250)	5*'*-GTTGAGGAAATCAAGGGTCATG-3*'*	5*'*-TGGACGATATTCTCCACATCC-3*'*
*ZP* (AT5G04340)	5*'*-TACGAAGGAAAGAACGGAGGC-3*'*	5*'*-GGTATCGGCGGTATGTTGAGG-3*'*
*CHLH* (AT5G13630)	5*'*-CAGCCAACATCAGTCTTGCT-3*'*	5*'*-ACCTGCTTCTTCTCAGCCAT-3*'*
*BAP1* (AT3G61190)	5*'*-ATCGGATCCCACCAGAGATTACGG-3*'*	5*'*-AATCTCGGCCTCCACAAACCAG-3*'*
*HEMA1* (AT1G58290)	5*'*-GTTGCTGCCAACAAAGAAGA-3*'*	5*'*-AATCCCTCCATGCTTCAAAC-3*'*
*PAL1* (AT2G37040)	5*'*-TTTTGGTGCTACTTCTCATCG-3*'*	5*'*-CTTGTTTCTTTCGTGCTTCC-3*'*
*PAL2* (AT2G37040)	5*'*-GTGCTACTTCTCACCGGAGA-3*'*	5*'*-TATTCCGGCGTTCAAAAATC-3*'*
*PAL3* (AT5G04230)	5*'*-CAACCAAACGCAACAGCA-3*'*	5*'*-CTCCAGGTGGCTCCCTTTTA-3*'*
*PAL4* (AT3G10340)	5′-GGTGCACTTCAAAATGAGCT-3′	5′-CAACGTGTGTGACGTGTCC-3*'*
*ACTIN2* (AT3G18780)	5′-AGCACTTGCACCAAGCAGCATG-3′	5′-ACGATTCCTGGACCTGCCTCATC-3′

### Determination of Chl content

The content of Chl was determined referring to the method described by Lichtenthaler^46^. Weighed segments of frozen crushed material (about 0.04 g) were homogenized in 1 mL 80% acetone and stood for 5–6 h, then centrifuged for 10 min at 5000 rpm at 4 °C and assayed spectrophotometrically at 663 nm and 645 nm. Result calculation: C_(mg/g)_ = (17.32 A_663_ + 7.18 A_645_)/m_(g)_. The experiment was performed in three independent biological repeats.

### AOPP treatment

Seeds were germinated on MS added with 1 mM Phe (SIGMA) and grown under SD. On the third day seedlings were sprayed with 100 μM AOPP (Wako) or ddH_2_O (as a control) once a day for 5 days, then the rate of death seedlings was counted, and the seedlings were photographed. The experiment was performed in three independent biological repeats.

### MeJA treatment

For determination of dead seedlings (Figs. 5 and 7), seeds were germinated on MS added without or with 0.5 mM Phe and/or 5 μM MeJA (SIGMA) and grown under SD for 6–8 days. For RT-qPCR analysis (Fig. 6), about 2-week-old seedlings growing under LD were transferred to SD and on the fourth day the seedlings were sprayed with 100 μM MeJA or ddH_2_O (as a control). After MeJA treatment for one day, the seedlings were harvested and used for RT-qPCR analysis. The experiment was performed in three independent biological repeats.

## Supplementary Information

Supplementary Figures.
