# Supplementary material for: Phenylalanine suppresses cell death caused by loss of fumarylacetoacetate hydrolase in Arabidopsis
Source: Sci Rep. 2022 Aug 8;12:13546. doi: 10.1038/s41598-022-17819-3 (PMC9360007; doi:10.1038/s41598-022-17819-3)
Supplement: Supplementary file 1 — Supplementary Figures. [file 41598_2022_17819_MOESM1_ESM.pdf]

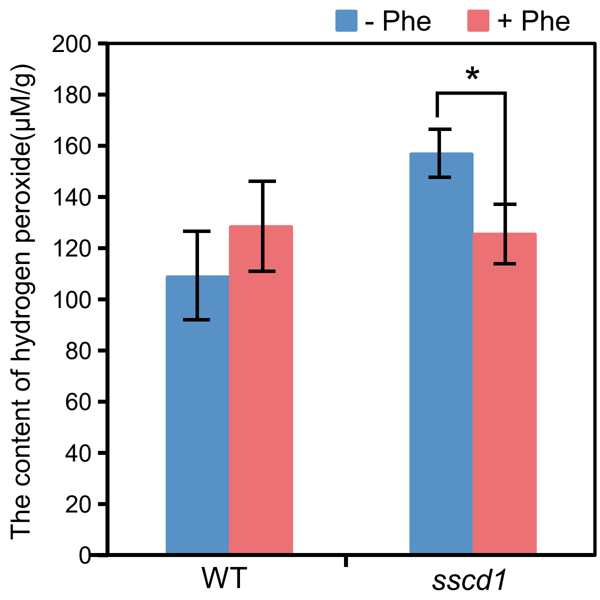

Fig. S1 The content of hydrogen peroxide in *sscd1* was significantly reduced after treatment with Phe.

The 7-d-old seedings of WT and *sscd1* grown on MS medium under LD were transferred to SD and sprayed with ddH_2O (-) or 2 mM Phe (+) once a day for 3 days, then the content of hydrogen peroxide in seedlings was determined. Each value is the mean of three independent biological replicates ± standard deviation. An asterisk represents the significance of differences (two-tailed Student's t-test) at the levels of $P<0.05$. Phe, Phenylalanine; WT, wild type, Col-0; LD, long day; SD, short day.

The content of hydrogen peroxide was determined referring to the ferrous oxidation-xylenol orange (FOX) assay (*J. Agric. Food Chem*. **50,** 248-254, 2002). The FOX reagent consisted 0.1 mM Dimethyl Phenol orange, 0.25 mM ferrous ammonia sulfate, 100 mM sorbitol, and 25 mM H_2SO_4. Weighed segments of frozen crushed material (about 0.05 g) were homogenized in 0.5 mL 25 mM H_2SO_4, then centrifuged for 10 min at 5000 rpm at 4 ℃. Took 100 μL of supernatant, added 900 μL of FOX reagent and mixed, stood for 1 hour, then assayed spectrophotometrically at 580 nm. Result calculation: $C_{(\mu M/g)} = (A_{580} - 0.003) / 0.155 / 0.2 / m_{(g)}$.

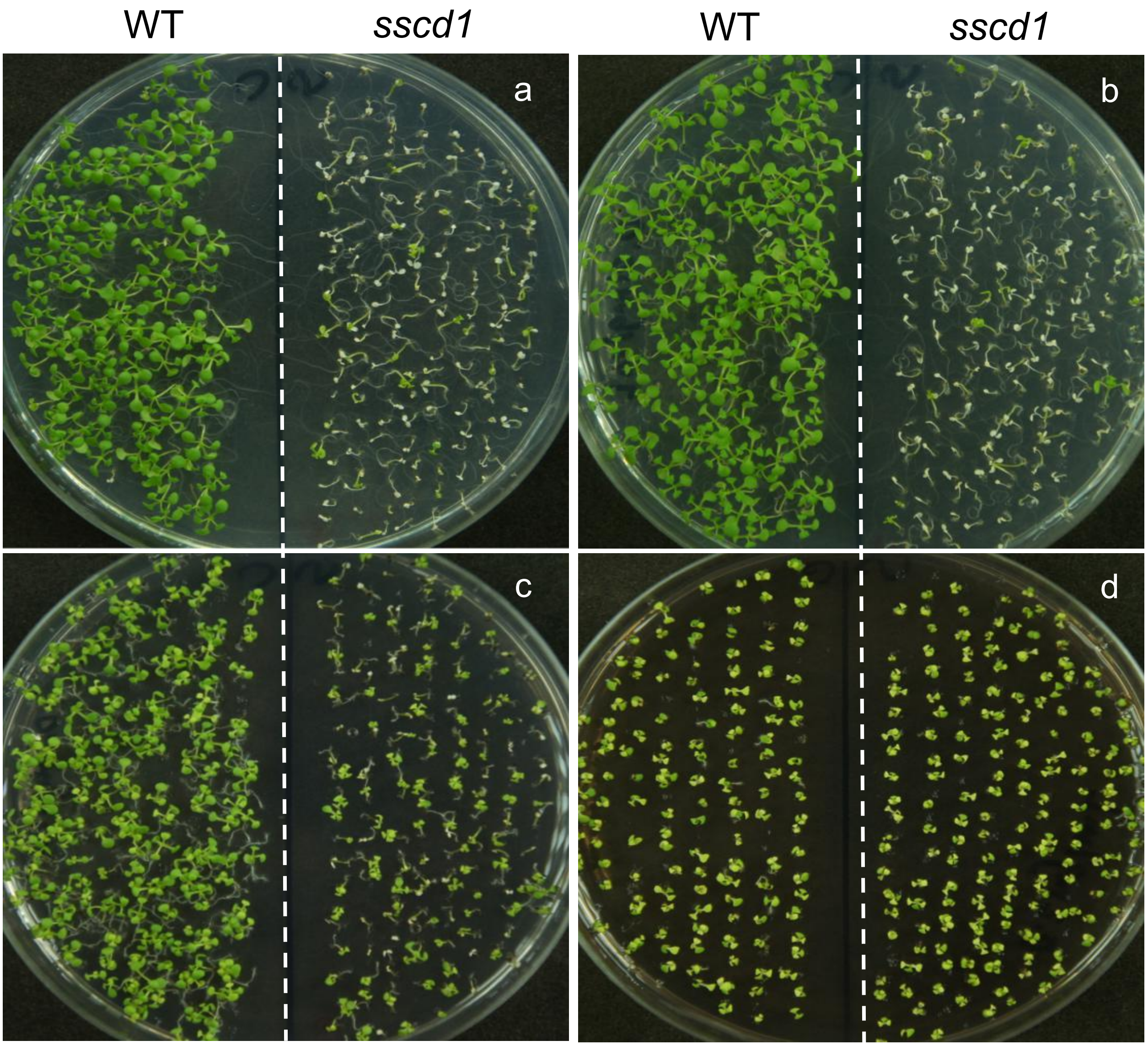

Fig. S2 Treatment with catechins suppressed the death of *sscd1* seedlings.

The phenotype of 14-d-old WT and *sscd1* seedlings grown on MS medium without (a) or with 0.01 (b), 0.1 (c), and 0.2 mg/mL (d) catechins under SD. WT, wild type, Col-0; SD, short day.

Seeds of WT and *sscd1* were plated on MS added without or with different concentrations catechins (0.01, 0.1, and 0.2 mg/mL) and grown under SD for 14 days, then the seedlings were photographed. The catechins at 0.1 mg/mL clearly reduced the death of *sscd1* seedlings (c). The catechins at 0.2 mg/mL severely inhibited the growth of WT seedlings (d), however, it completely suppressed the death of *sscd1* seedlings (d).
